# The Adsorption Mechanism of Hydrogen on FeO Crystal Surfaces: A Density Functional Theory Study

**DOI:** 10.3390/nano13142051

**Published:** 2023-07-11

**Authors:** Shujie Zhang, Kejiang Li, Yan Ma, Yushan Bu, Zeng Liang, Zonghao Yang, Jianliang Zhang

**Affiliations:** 1 School of Metallurgical and Ecological Engineering, University of Science and Technology Beijing, Beijing 100083, China; 2 Max-Planck-Institut für Eisenforschung, Max-Planck-Straße 1, 40237 Dusseldorf, Germany

**Keywords:** hydrogen metallurgy, iron oxides, hydrogen adsorption, density functional theory, transition states

## Abstract

The hydrogen-based direct reduction of iron ores is a disruptive routine used to mitigate the large amount of CO_2_ emissions produced by the steel industry. The reduction of iron oxides by H_2_ involves a variety of physicochemical phenomena from macroscopic to atomistic scales. Particularly at the atomistic scale, the underlying mechanisms of the interaction of hydrogen and iron oxides is not yet fully understood. In this study, density functional theory (DFT) was employed to investigate the adsorption behavior of hydrogen atoms and H_2_ on different crystal FeO surfaces to gain a fundamental understanding of the associated interfacial adsorption mechanisms. It was found that H_2_ molecules tend to be physically adsorbed on the top site of Fe atoms, while Fe atoms on the FeO surface act as active sites to catalyze H_2_ dissociation. The dissociated H atoms were found to prefer to be chemically bonded with surface O atoms. These results provide a new insight into the catalytic effect of the studied FeO surfaces, by showing that both Fe (catalytic site) and O (binding site) atoms contribute to the interaction between H_2_ and FeO surfaces.

## 1. Introduction

In primary iron- and steelmaking, the reduction of iron oxides by fossil fuels (such as coal and coke) generates a large amount of CO_2_ emissions, accounting for ~7% of global CO_2_ emissions [1,2,3]; it is considered to be a major cause of global warming. The use of hydrogen instead of carbon for iron oxide reduction has emerged as the most promising solution to mitigating CO2 emissions in the steelmaking industry. Among hydrogen-based reduction processes, hydrogen-based direct reduction currently possesses the highest technology readiness level (TRL: 6–8) and is readily employed at the industry level when a large amount of green hydrogen is available [4]. However, it was found that hydrogen-based direct reduction is very different from direct reduction using natural gas in terms of thermodynamics and kinetics [5,6,7]. Particularly, at the microscopic and atomistic scales, the underlying interaction mechanisms are not yet fully understood.

As early as the 20th century, the possibility of using H_2_ as a reducing agent in blast furnaces to reduce the use of carbon fuel in the ironmaking process was proposed [8,9,10]. Since then, the iron and steel industry [11,12,13] has developed rapidly with new processes of hydrogen-rich reduction [14,15] and hydrogen metallurgy [4,16,17,18,19]. These hydrogen-based metallurgical processes have been extensively studied both experimentally [20,21,22,23,24] and theoretically [5,6,25,26]. Raabe et al. summarized the hierarchical nature of the direct reduction of iron oxides by hydrogen at different scales [27], and the reduction mechanism of iron oxide by pure H_2_ was conducted on both macro- [28] and near-atomic scales with atomic probe tomography [29,30]. They also simulated the reduction using a chemo-mechanical phase field [31], demonstrating the significant influence of internal stress and micropores on iron oxide in hydrogen-based direct reduction process. Li et al. [32] reviewed the research progress on the reactivity of various hydrogens to oxides and emphasized the strong effect of oxygen vacancy on the surface of various hydrogens, and previous studies [33,34] have reached similar conclusions.

However, due to the limitations of existing experimental techniques, it is difficult to conduct further experiments on the atomic scale, so DFT [5,35,36,37,38,39,40] is often used to study the structure and properties of iron oxide [41,42,43,44] as well as the interaction between reducing gas and iron oxide. Wang et al. [45] used DFT to study the mechanism of CO reduction of Fe_2_O_3_ in the chemical cycle, which provided a significant theoretical solution for examining oxygen carrier materials and optimizing the microstructure of oxygen carriers. Menga et al. [25] studied the adsorption and dissociation of H_2_ on the surface of Fe_3_O_4_, the migration ability of the H atom, and the competitive relationship between H_2_ adsorption and deoxidation with the increase in the adsorption coverage of H. The reduction process of FeO is the most difficult and the last step in the reduction process of Fe_2_O_3_ to pure iron [46,47], so there are many studies that focus on the reduction of FeO. Lu et al. [5] studied the adsorption behavior of CO and H_2_ on different FeO surfaces and made a prediction of the main growth direction of metallic iron on FeO surfaces. However, excessive emphasis is placed on the comparison with CO and the magnetic properties of iron oxides have rarely been considered in previous calculations [5,6]. The adsorption mechanism of hydrogen atoms is not yet fully understood, and hydrogen dissociation is usually related to the catalysis of metal particles [48].

The interaction of H_2_ with FeO surfaces is important not only for iron making but also for heterogeneous catalytic reactions of FeO. In this paper, in order to have a deeper understanding of the hydrogen reduction mechanism at atomic scales, DFT was used to study the adsorption mechanism of H_2_ on the surface of different FeO crystals, and more accurate magnetic properties, +U [49], were used in the calculation. The influence of different surface atoms on the adsorption and dissociation of H_2_ was calculated and discussed, which is helpful for further experimental and theoretical research in the field of ferric oxide reduction or catalysis [50].

## 2. Computational Models and Methods

The Perdew–Burke–Ernzerhof (PBE) generalized gradient approximation (GGA) and the GGA + U method were applied to all calculations in this study, and the plane-wave-based DFT implemented in the open-source Quantum Espresso package was used in all simulations [51,52]. The kinetic energy cut-off wave function expansion was set at 90 Ry (1 Ry = 13.61 eV), while the charge density was set at 900 Ry according to our previous parametric tests [49]. To precisely describe the interactions between the atoms, DFT-D3 was adopted [53] to define the attraction between O and H. The magnetic moment of Fe was set to accurately characterize the FeO lattice structure, and U_Fe_ = 4 eV was set in order to have a better description of the orbitals of the transition metal Fe [49]. The convergence threshold for self-consistent calculations was set at 1 × 10^−5^ Ry. Relaxation calculations were performed using conjugate gradient minimization until the magnitude of the residual force on each atom was less than 1 × 10^−5^ Ry /Bohr and the total residual energy was in the range of 1 × 10^−5^ Ry with a k-point of 4 × 4 × 1 [54].

**Figure 1:**
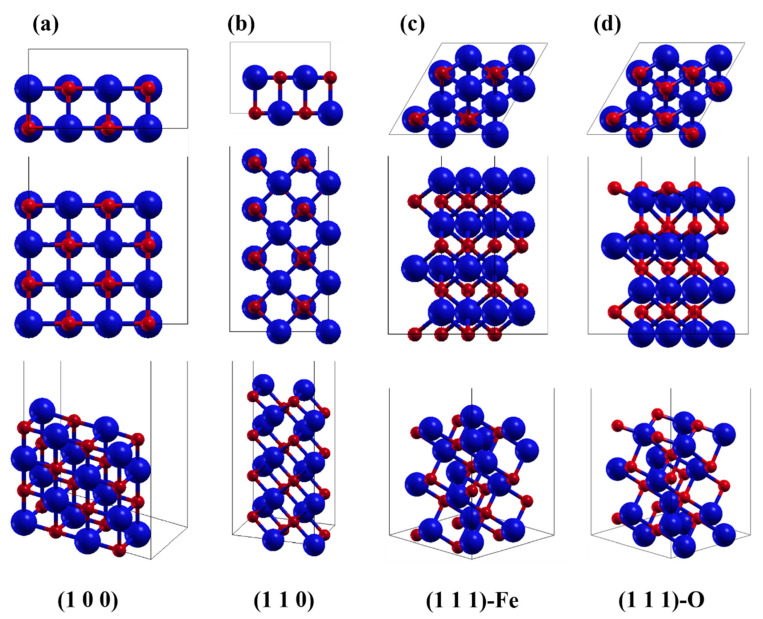
The investigated crystal surfaces of FeO: (**a**–**d**) (1 0 0), (1 1 0), (1 1 1) terminated with Fe, and (1 1 1) terminated with O, respectively. From top to bottom are the top view, the side view, and the three-dimensional stereogram. The red spheres are O atoms and the blue spheres are Fe atoms. The same holds for the following figures.

The individual surface structures of FeO are shown in Figure 1. To simulate the surfaces, all calculations were performed by relaxation calculations with half of the layers of atoms below being fixed to obtain the energy at equilibrium adsorption. The spin directions [25,55] are reversed layer by layer along the (100) direction according to our previous paper [49]. A total of four surfaces were used in this calculation, (100), (110), (111)-Fe, and (111)-O, which are common crystal surfaces for iron oxides [5].

### 2.1. Surface Energy Calculation

From a physical point of view, surface energy γ is composed of cleavage energy ($E_{cle}$) and relaxation energy ($E_{rel}$) [56,57], and surface energy is generally obtained by the following equation:

(1) $$\gamma =\left(E_{cle}+E_{rel}\right)/A$$

(2) $$E_{cle}=\left(E_{unrelax}-E_{bulk}\right)/2$$

where *A* is the surface area, $E_{unrelax}$ is unrelaxed energy, and $E_{bulk}$ is the energy of the bulk without the vacuum layer.

For a cut surface with symmetric ends, $E_{rel}$ can be easily obtained from the formula:

(3) $$E_{rel}=\left(E_{total}-E_{unrelax}\right)/2$$

However, for these asymmetric plates, it is difficult to calculate the relaxation energy of the upper and lower ends from Equation (3), which should be treated separately. The upper and lower surfaces after cutting are respectively called *T*_1_ and *T*_2_. The relaxation energy of *T*_1_ and *T*_2_ can be obtained by the following equations:

(4) $$E_{rel\left(T_{1}\right)}=\left(E_{T_{1}-relax}-E_{unrelax}\right)/2$$

(5) $$E_{rel\left(T_{2}\right)}=\left(E_{T_{2}-relax}-E_{unrelax}\right)/2$$

where $E_{T_{1}-relax}$ is the energy of a plate with only the upper half relaxed, and $E_{T_{2}-relax}$ is the energy of a surface with only the lower half relaxed. In this case, the relaxation section should be thick enough to avoid errors. The surfaces selected in this paper are asymmetric-type surfaces, so the surface energy can be calculated using the Equations (1), (2) and (4)/(5). The surface energy is obtained by dividing the difference between the total energy of the relaxed surface atoms ($E_{total}$) and the energy of the bulk without the vacuum layer ($E_{bulk}$) by twice the surface area (A).

### 2.2. Adsorption Energy Calculation

After calculating the surface relaxation, the energies of H_2_ molecules ($E_{H_{2}}$) and the surfaces of four kinds of iron oxides ($E_{sub}$) were calculated. Then, the adsorption energy *E_a_* between hydrogen (in the form of both *H*_2_ molecules and *H* atoms) and the iron oxide surface was calculated according to the following equation [58,59,60,61,62,63,64]:

(6) $$E_{a}{=E}_{sub+H_{2}}-{\;E}_{sub}-{\;E}_{H_{2}}$$

where $E_{sub+H_{2}}$ represents the total conformational energy of the *H*_2_ molecule adsorbed on the iron oxide surface, after relaxation calculations.

The adsorption energy (*E_a_*) also indicates the bonding strength between the iron oxide surface and the adsorbed hydrogen. The more negative the adsorption energy is, the stronger the adsorption will be. In the calculation of adsorption energy, *H*_2_ was placed 2.7 Å away from the surface as the initial state of adsorption, and the results are all indicative of physical adsorption. The initial adsorption state of H atoms was calculated according to the bonding length of different atoms, and the results indicate chemical adsorption.

### 2.3. Single-Point Energy Distribution Calculation

**Figure 2:**
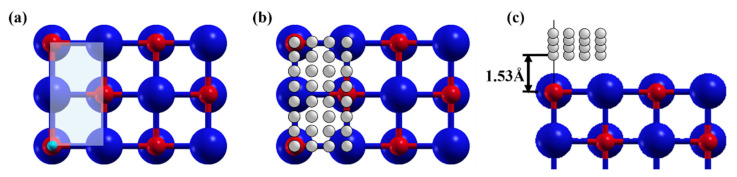
Schematic diagram of H atom selection region and arrangement. (**a**) is the periodic region diagram of the distribution of hydrogen atoms, (**b**) is the distribution diagram of hydrogen atoms in the same layer, and (**c**) is the distribution diagram of hydrogen atoms in different layers on the surface, and the layer spacing is 0.31 Å.

The single-point energies of H at different positions on different surfaces were calculated, formulating a two-dimensional energy surface. In order to reduce the cost and repeatability of calculations, the smallest units of the supercell were selected as the main distribution area of H, as shown in Figure 2a. Since the diameter of a H atom is 0.62 Å, the H atoms are arranged at a distance close to 0.62 Å in the horizontal direction to ensure that the H atoms are presented at all important points in this region (four corners of the region), as shown in Figure 2b. In the vertical direction, the position of H atoms in each layer is set at intervals equal to the radius of a H atom. The lowest place was D = 1.53 Å from the surface (approximate distance of H–Fe bond length, used in order to prevent atoms from being too close and to avoid errors), as shown in Figure 2c. After the points were determined, the script was used to generate files in batches and submit them for calculation and statistics. Finally, the horizontal and vertical coordinates of H were taken as x and y axes, energy was concluded as a cloud map, and different D heights were taken as the z axis.

### 2.4. Transition State Calculation

The NEB (nudged elastic band) allows the calculation of its reaction path or adsorption potential in the transition state. In this case, the neb.x module of Quantum Espresso software was used for the transition state search. Six transition configurations (plus beginning and end states) were expected to reduce the computational cost. The kinetic energy cut-off wave function expansion was set to 55 Ry (1 Ry = 13.61 eV), and the charge density was set at 600 Ry, and the k-point was 3 × 3 × 1.

## 3. Results and Discussion

### 3.1. Crystal Surface Energy Analysis

The calculated surface energy of FeO is summarized in Table 1 (see Table S1 for detailed data). The lowest energy of the (100)-FeO surface (i.e., 0.6871 J/m^2^) suggests that it is the most stable surface, while the (111)-FeO surface has the highest energy and it is prone to reactions. This trend is in good agreement with the results reported in the literature. Meng [65] et al. investigated the dependence of FeO surface energy on γ, and found that (100)-FeO surfaces are more stable than (110) surfaces under the condition of PBE + U. Although there is a numerical difference, it is believed that the difference is dependent on whether the magnetic force is set.

**Figure 3:**
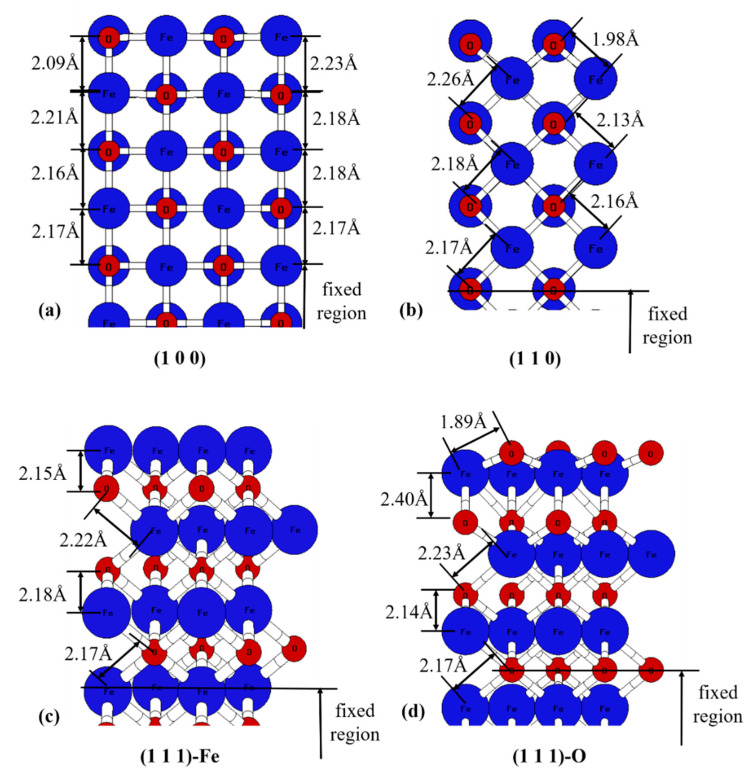
Surfacereconstruction for FeO crystal (side view): (**a**–**d**) shows the results for (1 0 0), (1 1 0), (1 1 1)-Fe, and (1 1 1)-O, respectively.

The length of Fe–O bonds after surface relaxation is shown in Figure 3. When the Fe atom is exposed at the top of the (100) surface (2.23 Å), the Fe–O bond is slightly longer than when the O atom is at the top of the (100) surface (2.09 Å). With the exception of (1 0 0), the bonds between the first and second layers are generally shortened, while the bonds between the second and third layers are elongated. The bonding length of the top layer of the surface is shortened by about 0.02 to 0.283 Å, while the bonding length of the second layer is extended. The surface relaxation extent of (111)-Fe and (111)-O are very different due to the difference of the surface atoms since surface O is more active compared with surface Fe.

### 3.2. Distribution of Surface Energy with a Single H Atom on Top of the FeO Surfaces

**Figure 4:**
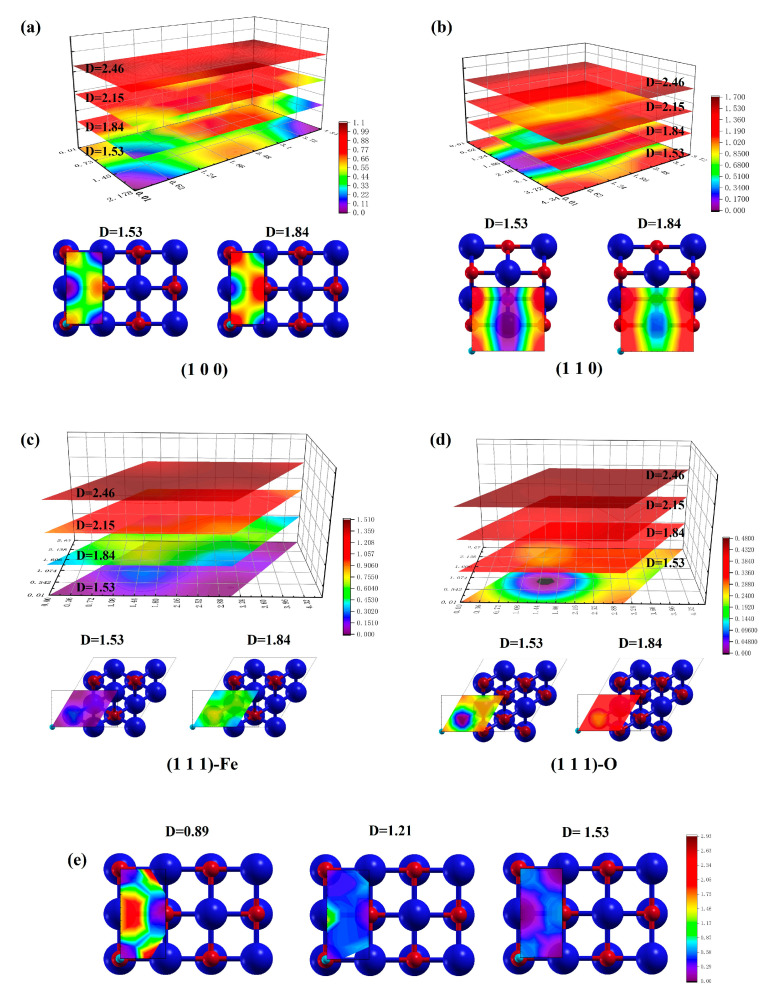
Energy distribution with H atom located at different sites above the crystal surface: (**a**–**d**) shows the results for (1 0 0), (1 1 0), (1 1 1)-Fe, and (1 1 1)-O surface, respectively. (**e**) shows the results of calculations for (1 0 0) surfaces closer to the surface, and it is different from the scale of (**a**). The horizontal and vertical coordinates are atomic coordinates, and D is the distance between H and the surface. The unit is Å.

The distribution of surface energy with a single H atom placed at different sites on top of the FeO surfaces was computed and the result is shown in Figure 4. In Figure 4a,b, the energy distribution near the position of the O or Fe atom is significantly different among the four configurations. In Figure 4c, the results are almost correlated with distance D, indicating that each layer has its own continuous and tight energy differentiation. This result indicates that different atoms on the surface have obvious influences on adsorption, and the energy distribution is more continuous near the Fe atom, while O is only concentrated in a very close area to have stable adsorption.

This observation can also be attributed to the different atomic radii or potential fields of Fe and O atoms. The bonding distance between H and Fe is close to 1.53 Å, exactly the distance closest to the plane where the calculations were performed. It is possible that this distance is not within the range of obvious influence of the O atom, as the common O–H bond length is 0.9–1.0 Å [66] In a previous calculation of H bonding on FeO surfaces, the bond length was also found to be between 0.98–1.03 Å [5]. Therefore, more calculations were performed to verify this hypothesis, as shown in Figure 4e, which is the same single-point energy calculation, except that it is closer to the plane. After drawing closer to the plane, there is a gradual decrease in energy near O and a rapid increase in energy near the Fe atom. This result indicates that H is very close to Fe at this point, showing that the different radii of the Fe and O atoms affect H adsorption. The atomic radius of O is smaller than that of Fe, so when H is at the same distance from the surface, the energy near O atoms is higher than that near Fe atoms.

### 3.3. Bonding Adsorption of H on FeO Surface Site

**Figure 5:**
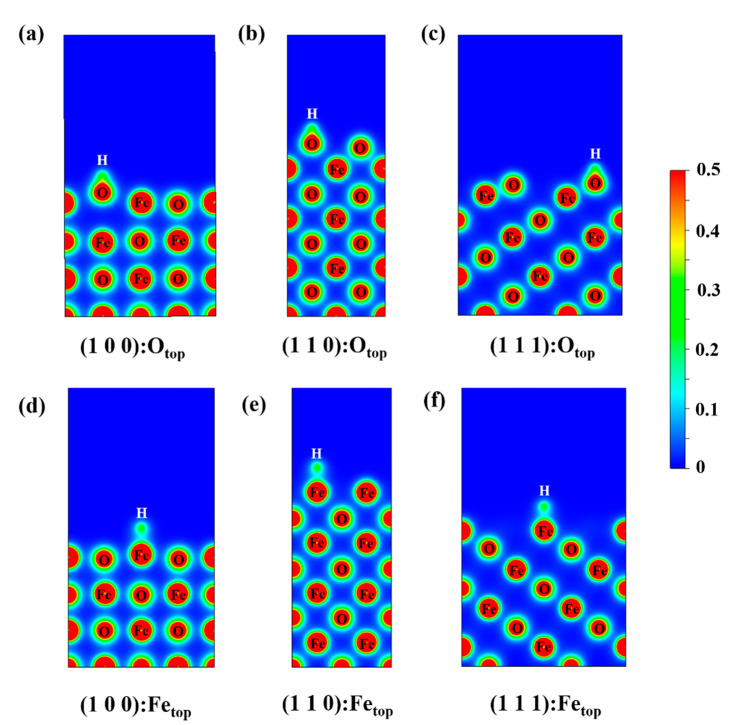
Charge density distribution: (**a**–**c**) is the charge density distribution of H adsorbed at O_top_ position on the (1 0 0), (1 1 0), (1 1 1)-O surfaces of FeO. (**d**–**f**) is the charge density distribution of H adsorbed at the Fe_top_ position on the (1 0 0), (1 1 0), (1 1 1)-Fe surface of FeO. The equivalent surface is 0.5 e/A.

Relaxation calculations were carried out to allow the sole H atoms to be bonded at the top sites of O and Fe, and the adsorption energies and differential charge density plots were calculated and are summarized in Table 2. All energies are negative and they are lower at O_top_ than those at Fe_top_, indicating that hydrogen adsorbed on O_top_ is more stable. The bond lengths are similar for each condition, and the H–Fe lengths coincide with the height of the low-energy region of H near the FeO surface sites (Section 3.2), while the O–H bond lengths are similar to those in Figure 4e. The charge distribution of H adsorbed on the surface is shown in Figure 5. Obviously, the H–O electron is stable and the bond is short when adsorbed near the O site, and the electron density between Fe and H is much lower compared with that between O and H.

**Figure 6:**
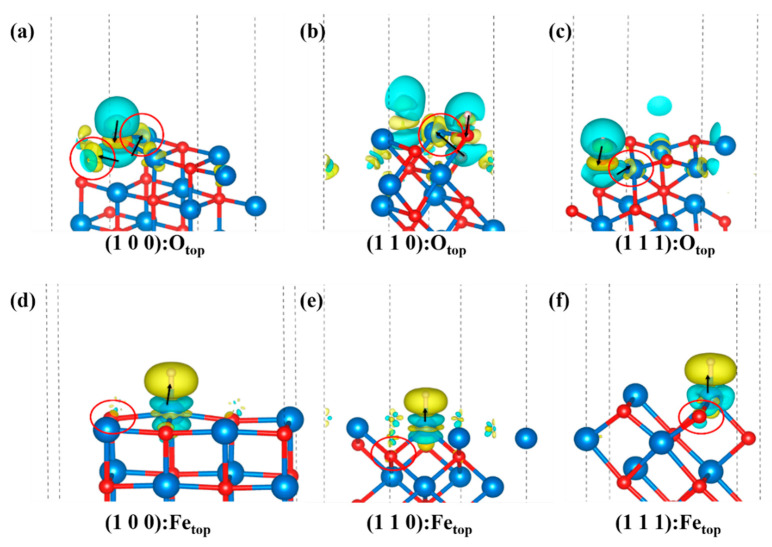
Differential charge density diagram: (**a**–**c**) is the differential charge density diagram of H adsorbed at O_top_ position on the (1 0 0), (1 1 0), (1 1 1)-O surfaces of FeO. (**d**–**f**) is the differential charge density diagram of H adsorbed at the Fe_top_ position on the (1 0 0), (1 1 0), (1 1 1)-Fe surface of FeO. Blue is electron loss, yellow is electron gain, red circles show electron gain and loss for atoms not directly bonded, and arrows point to the main direction of charge transfer. The isosurface is 3 × 10^−5^ e/Å.

In addition, the differential charge density diagram in Figure 6 shows that H gains electrons when bonded to Fe and loses electrons when bonded to O. The adsorption on O has effects on the surrounding electrons on Fe, but there is only a weak influence if it is adsorbed on Fe. This fact suggests that H prefers to bond with O and further supports the hypothesis that the small radius of the O atom prevents bonding at the same distance, making it more energetically favorable.

### 3.4. Hydrogen Adsorption on FeO Surfaces

**Figure 7:**
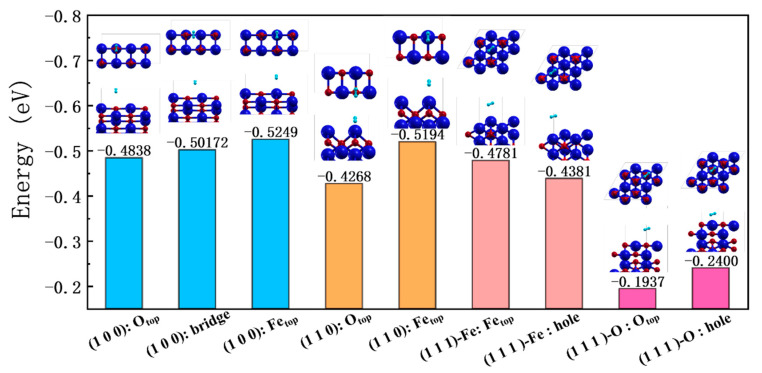
The adsorption of H_2_ at different positions on each surface of FeO. Different surfaces are marked with different colors. In each small diagram, the top half is a top view and the bottom half is a three-dimensional view.

The adsorption of H_2_ at different positions on each FeO surface was investigated and the results are shown in Figure 7. Fe_top_’s results are consistent with those of Li [67] et al. In addition to H_2_ horizontal to the surface [68], Table 3 shows the results for the configuration of H_2_ perpendicular to the surface for reference. Considering all the calculations, we find that, in any plane, H_2_ has stronger physical adsorption at the Fe top site, while the weakest adsorption occurs at the O top site. The adsorption energies of the different planes are basically in the range of 0.45–0.5 eV, which is the range for physical adsorption, except for the (1 1 1)-O surface, for which the result differs from the others by almost half of the energies of other surfaces. This is probably because H_2_ does not bond to any of the atoms on the surface, preferring to stick to Fe. However, the top layer of the (1 1 1)-O surface is full of O atoms, which is not conducive to H_2_ adsorption.

**Figure 8:**
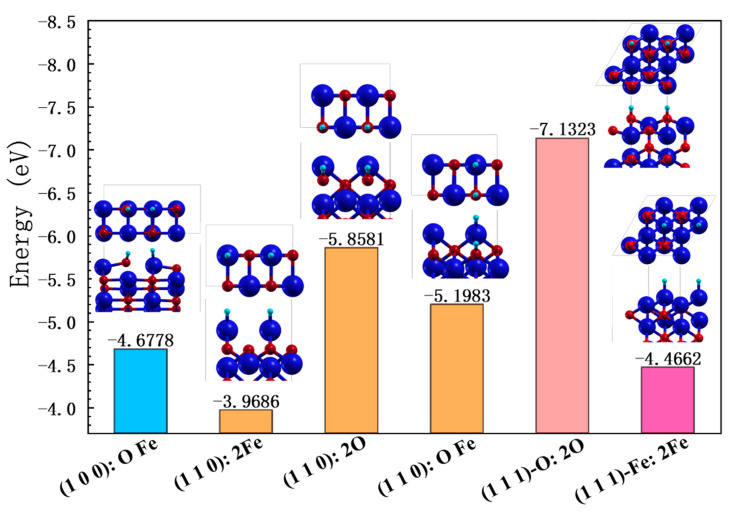
Adsorption of two H atoms at different positions on each surface of FeO. Different surfaces are marked with different colors. In each small figure, the top half is a top view and the bottom half is a three-dimensional view.

Secondly, we also calculated the possible adsorption modes of the two H atoms after the dissociation of H_2_ on the surface, as shown in Figure 8. The findings show that both H atoms bonded to O atoms result in more stable adsorption, while adsorption on both Fe atoms is the least stable adsorption case. This result is consistent with the observation in Section 3.2, as H bonded to O is energetically more favorable. A comparison of the adsorption on different surfaces shows that the adsorption of the two H atoms is related to the position and the activity of the surface atoms. On the (100) surface, the two H atoms are adsorbed on Fe and O, while on the (110) surface, the structure is more stable, due to the fact that the uppermost atoms on the (110) surface are less bonded to the lower layer. The more active surface atoms of the (1 1 1) surface can also be validated from the hydrogen adsorption energy on the double O or double Fe sites of the (1 1 0) and (1 1 1) surfaces. This is in line with the results for the surface energy.

In summary, H_2_ prefers to adsorb on the Fe_top_ site. The same conclusion was also obtained in the calculation of molecular dynamics by Cheng [69] et al. This conclusion coincides with the results of the H energy cloud diagram, which suggests that physisorption on the surface relies mainly on the attraction capacity of Fe, which is particularly evident on the (111)-O surface. The adsorption of two H atoms on the surface shows that H prefers to bond with O [70] and that the adsorption is also related to whether the surface is active or not.

### 3.5. H_2_ Dissociation and Adsorption on FeO Surfaces

**Figure 9:**
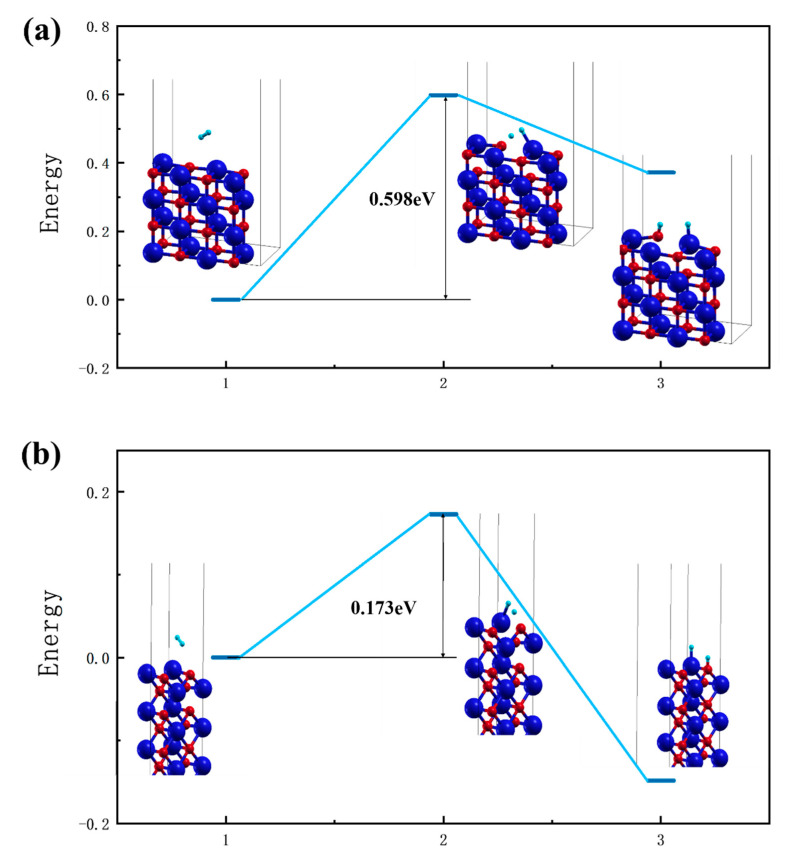
(**a**) and (**b**) are the calculated results of the dissociation and bonding transition states of H_2_ adsorbed on FeO(100) and (110) surfaces, respectively. Labels 1, 2, and 3 represent the initial state, the transition state, and the final state.

These results suggest that when H_2_ is likely to react with FeO, it first physically adsorbs on Fe and then dissociates into two H atoms, forming a chemisorption bond with the atoms on the surface. The exact atom on which these two H atoms adsorb needs to be considered in terms of the specific surface and the temperature, as the mass of an H atom is too small, so it can vibrate around very easily and may also become free again. Based on this conjecture, the dissociation adsorptions of H_2_ on the FeO (100) and (110) surfaces were calculated by using the NEB method, as shown in Figure 9. The initial and final states in the figure show the results of the relaxation calculations.

The dissociation and adsorption processes of H_2_ on the FeO surfaces are summarized as follows: physical adsorption of H_2_ on top of a Fe atom; dissociation into two H atoms; and formation of chemisorption, where the bonding atoms are pulled out to a small extent to allow subsequent reactions to occur. For the (100) surface, the dissociative adsorption of H_2_ molecules only needs to cross an energy barrier of 0.598 eV, while for the higher, more active (110) surface, a maximum energy barrier of 0.173 eV is required to complete the dissociation of H_2_ molecules. This is consistent with the results of Wang [71] et al. (The calculation of transition states has also been attempted for the (111) surface. However, due to the large distance between the O/Fe atoms on the surface and the more active surface and the small size of the H atoms, it is easy to migrate into the block to form the block OH and hydride [32]).

The calculated bond energy of H–H is 4.53 eV, which is consistent with the value of 4.48 eV reported in the literature [72]. H_2_ alone requires a lot of energy to dissociate, but the adsorption with Fe atoms and bonding with O atoms on the surface of FeO make dissociation easier. This is the catalytic action of H_2_ [73] on the surface of Fe [70], and the same metallic catalysis also occurs on other metals. For example, H_2_ dissociation on Pb requires only 0.06 eV [68]. Also, different doping atoms on the Mg surface can catalyze H_2_ dissociation, and all of them have bond energies below 1.15 eV, far lower than the H_2_ bond energy of 4.48 eV. Ce_2_O_3_ can even make the dissociation energy barrier of H as low as ~0.1 eV [74]. The study of Nobuhara et al. [75] also shows that the energy barrier of H_2_ dissociation and adsorption on Ti, Ni, Pd, and La surfaces is very small or negligible. At the same time, the strong influence of oxygen vacancy on the stability and reactivity of various hydrogens on the oxide surface has been reported in previous studies [32,73]. For FeO surfaces, the Fe position on the surface is also an O vacancy. On FeO surfaces, H is always present in the form of the hydroxyl group. The presence of oxygen vacancy inhibits the production of water but favors the production of H_2_. This explains why H_2_ is bonded to O even though it dissociates around Fe.

## 4. Summary

In this study, the adsorption of H_2_ molecules and H atoms on different FeO surfaces has been calculated by density functional theory (DFT) to explore the adsorption behavior of H2 on different FeO surfaces. Through comparative analysis, the following conclusions were obtained:

During chemisorption, H atoms tend to bond with O on the surface to maintain stability, but H_2_ molecules are more inclined to adsorb on the top of Fe on the FeO surface when physical adsorption is performed.

The behavior of H atoms on the surface can be attributed to the charge distribution range (or effective atomic radius) of surface atoms. The effective atomic radius of Fe is much larger than that of O. When O and Fe are alternately arranged, H_2_ is mainly attracted by the surface Fe atoms. After the dissociation of H_2_ molecules into H atoms, the active H atoms are captured in a more strongly bonded manner by O on the surface.

After being attracted to Fe on the surface to form physical adsorption, H_2_ molecules were activated by surface Fe and O and then passed through a small dissociation energy barrier of only 0.173 eV and eventually bonded to form chemisorption on the surrounding surface atoms with a tendency to adsorb on O.

This study provides an understanding of the mechanisms of H_2_ dissociation on metal oxide surfaces and hydrogen adsorption dissociation, and the effects of different surface atoms on H_2_ at different stages of the surface reaction were discussed, which is highly relevant in the study of sustainable metallurgical processes using hydrogen.

## Figures and Tables

**Table 1:** Surface energy of individual crystallographic surfaces of FeO crystal.

Surface	(1 0 0)	(1 1 0)	(1 1 1)-Fe	(1 1 1)-O
Surface energy (J/m^2^)	0.6871	1.4070	2.3993	1.3817

**Table 2:** Adsorption energies of H atoms on each FeO surface when adsorbed at the top Fe and O positions.

	Surface	Energy/eV	H Bonding Length/Å
Fe_top_	(1 0 0)	−1.8288	1.582
	(1 1 0)	−2.4052	1.562
	(1 1 1)-Fe	−2.2609	1.557
O_top_	(1 0 0)	−2.7816	0.982
	(1 1 0)	−3.0680	0.979
	(1 1 1)-O	−3.7424	0.972

**Table 3:** Adsorption energies for H_2_ adsorption on individual surfaces, including results perpendicular to the surface, in eV.

	Horizontal				Perpendicular	
	H_2_-Fe_top_	H_2_-O_top_	Bridge	Hole	H_2_-Fe_top_	H_2_-O_top_
(1 0 0)	−0.5249	−0.4838	−0.5017		−0.4957	−0.5036
(1 1 0)	−0.5194	−0.4268			−0.4854	−0.4607
(1 1 1)-O		−0.1937		−0.2400		−0.2167
(1 1 1)-Fe	−0.4781			−0.4381	−0.4660	

## Data Availability

The data that support the findings of this study are available from the corresponding author upon reasonable request.

## Supplementary Materials

The following supporting information can be downloaded at: https://www.mdpi.com/article/10.3390/nano13142051/s1, Table S1: Surface energy test results. The atoms that fix half the layers 23 of the cell from the bottom up.
