# Supplementary material for: The Adsorption Mechanism of Hydrogen on FeO Crystal Surfaces: A Density Functional Theory Study
Source: Nanomaterials (Basel). 2023 Jul 11;13(14):2051. doi: 10.3390/nano13142051 (PMC10384720; doi:10.3390/nano13142051)
Supplement: Supplementary file 1 [file nanomaterials-13-02051-s001.zip › nanomaterials-2477447-supplementary.pdf]

Supplementary Materials

The Adsorption Mechanism of Hydrogen on FeO Crystal Surfaces: A Density Functional Theory Study

Shujie Zhang [1], Kejiang Li [1,*], Yan Ma [2], Yushan Bu [1], Zeng Liang [1], Zonghao Yang [1] and Jianliang Zhang [1]

[1] School of Metallurgical and Ecological Engineering, University of Science and Technology Beijing, Beijing 100083, China; shujiez@126.com (S.Z.); onion011213@163.com (Z.Y.)
[2] Max-Planck-Institut für Eisenforschung, Max-Planck-Straße 1, 40237 Dusseldorf, Germany
* Correspondence: likejiang@ustb.edu.cn

Table S1. Surface energy test results. The atoms that fix half the layers of the cell from the bottom up.

	total number of layers	Surface energy(J/m^2)
100	4	0.6835
	8	0.6854
	10	0.6871
110	6	1.3826
	8	1.4048
	12	1.4070
111-Fe	6	2.4044
	12	2.3993
111-O	6	1.3975
	12	1.3817
